# Building better interdisciplinary scientists: creating graduate level courses to address the communication gap in interdisciplinary research

**DOI:** 10.1186/1471-2105-13-S12-A9

**Published:** 2012-07-31

**Authors:** Denise R Koessler, Elizabeth G Johnson, Jordan M Utley, Harry A Richards, Cynthia B Peterson

**Affiliations:** 1 Department of Electrical Engineering and Computer Science, The University of Tennessee, Knoxville, TN 37996, USA; 2 Department of Microbiology, The University of Tennessee, Knoxville, TN 37996, USA; 3 UT/ORNL Graduate School of Genome Science and Technology, The University of Tennessee, Knoxville, TN 37996, USA; 4 SCALE-IT Program Administrator, The University of Tennessee, Knoxville, TN 37996, USA; 5 Department of Biochemistry and Molecular Biology, The University of Tennessee, Knoxville, TN 37996, USA

## Background

The SCALE-IT (**S**calable **C**omputing **a**nd **L**eading **E**dge **I**nnovative **T**echnologies) program at the University of Tennessee-Knoxville is one of an increasing number of programs at institutions across the country that relies on the success of interdisciplinary research. To prepare students for interdisciplinary problem solving, universities typically offer advanced courses or seminars in interdisciplinary topics. While courses like this are ideal for advanced students who have extensive backgrounds in both computational science and domain sciences, most graduate students lack core competency in fields outside of their own disciplines and are thus unprepared to step up to high-level multidisciplinary courses.

However, traditional institutional curricula do not provide opportunities for graduate students to develop an appropriate ground-level understanding in disciplines outside of their primary department. To address this issue, the SCALE-IT program has initiated the creation of introductory graduate level courses to overcome the lexical barrier between academic fields. Over the past two years, the SCALE-IT program has developed four successful graduate level courses dedicated to teaching introductory topics in bioinformatics at the University of Tennessee.

## Results

One course in particular, A Survey of Biology for Computational Researchers, demonstrates the success of this SCALE-IT initiative. This course aims to introduce a survey of biology to graduate students in other computational fields by addressing the crippling language barrier and building a community around six computational topics. The six topics explored are: Genomics, Biochemistry and Protein Biophysics, Cell Biology and Cell Signaling, Immunology, Phylogenetics and Evolution, and Populations Ecology. During the course of the semester, each topic concludes with a guest lecture and open discussion from an expert at the University of Tennessee in the computational domain. Graduate students from across five different academic disciplines are registered in this course for its first semester of instruction.

## Conclusions

In response to the high level of interest and success in these courses at the University of Tennessee, SCALE-IT is working towards establishing this curriculum as a permanent foundation in graduate interdisciplinary education. The permanent inclusion of graduate level basic courses would greatly enhance the versatility of the interdisciplinary student. In turn, the development of more capable graduate students will directly enable university-level academic fields to make greater strides in advancing the scope of successful interdisciplinary research.

